# Anti-Oxidant and Anti-Diabetes Potential of Water-Soluble Chitosan–Glucose Derivatives Produced by Maillard Reaction

**DOI:** 10.3390/polym11101714

**Published:** 2019-10-18

**Authors:** Thi Ngoc Tran, Chien Thang Doan, Van Bon Nguyen, Anh Dzung Nguyen, San-Lang Wang

**Affiliations:** 1 Department of Chemical and Materials Engineering, Tamkang University, New Taipei City 25137, Taiwan; tranngoctnu@gmail.com; 2 Department of Chemistry, Tamkang University, New Taipei City 25137, Taiwan; doanthng@gmail.com; 3 Doctoral Program in Applied Sciences, College of Science, Tamkang University, New Taipei City 25137, Taiwan; 4 Department of Science and Technology, Tay Nguyen University, Buon Ma Thuot 630000, Vietnam; bondhtn@gmail.com; 5 Institute of Biotechnology and Environment, Tay Nguyen University, Buon Ma Thuot 630000, Vietnam; nadzungtaynguyenuni@yahoo.com.vn; 6 Life Science Development Center, Tamkang University, New Taipei City 25137, Taiwan

**Keywords:** chitosan–glucose derivatives, Maillard reaction, anti-oxidant, anti-α-amylase, anti-α-glucosidase, anti-diabetes

## Abstract

Chitosan-sugar derivatives demonstrate some useful biology activities (for example anti-oxidant and anti-microbial activities). In this study, water-soluble chitosan–glucose derivatives (WSCGDs) were produced from a water-soluble chitosan hydrochloride (WSC) with 12.5 kDa of molecular weight and 24.05% of degree of acetylation (DA) via Maillard reaction with the heating temperatures of 100 °C and 121 °C. The Maillard reaction between WSC and glucose was investigated by measuring the absorbances at 420 nm and 294 nm, indicating that the reaction took place more effectively at 121 °C. All WSCGDs exhibited higher anti-oxidant activity than WSC, in which WSCGDs obtained at the treatment 121 °C for 2 h, 3 h, and 4 h expressed the highest ability (IC50 range from 1.90–1.05 mg/mL). Increased anti-α-amylase and anti-α-glucosidase activities were also observed in WSCGDs from the treatment at 121 °C. In detail, the highest IC50 values of anti-α-amylase activity were 18.02 mg/mL (121 °C, 3 h) and 18.37 mg/mL (121 °C, 4 h), whereas the highest IC50 values of anti-α-glucosidase activity were in range of 7.09–5.72 mg/mL (121 °C, for 1–4 h). According to the results, WSCGD obtained from 121 °C for 3 h was selected for further characterizing by high performance liquid chromatography size exclusion chromatography (HPLC SEC), colloid titration, FTIR, as well as 1H-NMR, indicating that the derivative of WSC and glucose was successfully synthesized with a molecular weight of 15.1 kDa and degree of substitution (DS) of 34.62 ± 2.78%. By expressing the excellent anti-oxidant and anti-diabetes activities, WSCGDs may have potential use in health food or medicine applications.

## 1. Introduction

Chitosan is a straight-chain polymer produced from the deacetylation process of chitin—the essential component of fungal cell wall, arthropod exoskeleton, and squid pen [1,2,3]. As a result of exhibiting numerous interesting bio-activities, chitosan is considered as potential material for applications in different fields, such as in biotechnology, environment, nutraceutical food, agriculture, or medicine [2,3,4,5,6,7,8,9,10]. However, the poor water-solubility of chitosan has caused obstacles for its applications. Therefore, water-soluble chitosan (WSC)—a modification of chitosan with higher solubility in neutral pH solution—also attracts great attention from many researchers. WSC can be obtained from chitosan in different ways, including shortening chitosan chains, increasing the degree of deacetylation or introducing hydrophilic groups into chitosan molecules So far, WSC demonstrates numerous activities, such as anti-cancer, anti-oxidant, or anti-microbial [11,12,13,14].

The Maillard reaction is an addition reaction commonly appearing in food processing, in which an amine group of nitrogenous compound condenses with a carbonyl group of reducing sugar. By introducing a high number of amine groups, chitosan also expresses the ability to condense with carbonyl groups of reducing sugars [15,16,17,18,19]. In fact, the chitosan–sugar derivatives produced by Maillard reaction reveal enhanced anti-oxidant or anti-microbial activities. However, there are few reports on the bio-activities of water-soluble chitosan–glucose derivatives (WSCGDs) [20,21,22,23]. Additionally, the conditions for performing Maillard reactions, as well as water-soluble chitosan sources in those reports also differ. Consequently, producing WSCGDs and investigating their bio-activities is of interest.

Type 2 diabetes (caused by insulin resistance) accounts for over 90% of the total cases of diabetes disease [24]. Inhibiting the activity of α-amylase and α-glucosidase in the digestive system is an effective therapy for managing type 2 diabetes. Consequently, numerous natural compounds (mainly produced by plants and microorganisms), as well as synthetic compounds, which show anti-α-amylase or anti-α-glucosidase activity, have been reported [24,25,26,27,28,29,30,31,32,33,34,35,36,37,38,39,40]. In addition, several reports reveal that chitosan and its derivatives also possess anti-diabetes activity, including anti-α-amylase or anti-α-glucosidase ability, making it a potential candidate for anti-diabetic drug application [41,42,43]. Additionally, there is no report on the anti-diabetes activity of WSCGDs, promising a new contribution of this study in the related fields.

In order to find the useful activities of chitosan derivatives, a 12.5 kDa WSC was used to produce WSCGDs via Maillard reaction herein. The reaction was determined and confirmed by the measurements of the absorbances at the 420 nm and 294 nm wavelengths. The anti-oxidant and anti-diabetes (anti-α-amylase and anti-α-glucosidase) activities of WSCGDs were investigated and also compared with native WSC. Additionally, further characterization by high performance liquid chromatography size exclusion chromatography (HPLC SEC), FTIR, colloid titration and ^1^H-NMR analysis was conducted to confirm the conjugation reaction.

## 2. Materials and Methods

### 2.1. Materials

Water-soluble chitosan hydrochloride (WSC) with 12.5 kDa of molecular weight and 24.05% of degree of acetylation (DA) was provided by the Microorganisms and Biochemistry Laboratory, Life Science Development Center, Department of Chemistry, Tamkang University (New Taipei, Taiwan). The major monomer components of WSC were glucosamine and *N*-acetyl-glucosanine (Figure S1) analyzing by enzyme method using chitinase from *Streptomyces speibonae* TKU048 [44]. The solubility of WSC was shown in Figure S2, which expressed the soluble capacity until pH 7.2. Before being used, the WSC was dissolved in distilled water at 5 mg/mL; the solution was then dialyzed against water for 3 d using a 10 kDa cut-off membrane for washing. Later on, WSC was concentrated from the solution by lyophilization method. The enzymes (yeast α-glucosidase and pork α-amylase), 3,5-dinitrosalicylic acid (DNS), *p*-nitrophenyl-α-d-glucopyranoside (*p*NPG), toluidine blue, 2,2-diphenyl-1-picrylhydrazyl (DPPH) were all bought from Sigma-Aldrich (Taipei, Taiwan). KS-802 column was purchased from Showa Denko K.K. (Tokyo, Japan).

### 2.2. Synthesis of Water-Soluble Chitosan–Glucose Derivatives

A stock solution of 2 mg/mL WSC and 2 mg/mL glucose in distilled water was prepared for the reaction. Consequently, the Maillard reaction was performed at 100 °C (using a water bath) or 121 °C (using an autoclave) for 1–4 h. The samples were then dialyzed against water for 3 d to remove the residual glucose, and dried by a lyophilizator. A spectrophotometer(Bio-Rad, Taipei, Taiwan) was used to measure the absorbance at wavelengths 294 and 420 nm of WSCGDs solution to determine the Maillard reaction.

### 2.3. Anti-Oxidant Activity Assay

The DPPH radical scavenging activity method was used to test the anti-oxidant activity of WSCGDs [41]. Briefly, 250 µl DPPH solution (1 mM in methanol) was mixed with 50 µL WSCGDs solution. Later on, the mixtures were kept at 20 °C in the dark for 20 min. The violet level of the solution was measured at a wavelength of 517 nm. The blank was prepared by a similar procedure except that 50 µL WSCGDs solution was replaced by 50 µL distilled water. The DPPH radical scavenging activity was calculated by the following Equation (1):

Activity (%) = (A_517_ of the blank − A_517_ of the sample)/A_517_ of the blank × 100 (1)

### 2.4. Anti-α-Amylase Activity Assay

Anti-α-amylase activity of WSCGDs was measured by the method of Nguyen et al. [45], with some modifications. Briefly, the 100 µL sample (WSC or WSCGDs solution) was mixed with 100 µL α-amylase solution (2 U, prepared in 0.1 M Tris-HCl buffer, pH 7) and kept at 20 °C for 30 min. The residual activity of α-amylase in the mixture was determined by the DNS method. The anti-α-amylase activity was calculated by the following Equation (2):

Activity (%) = (A_540_ of the blank − A_540_ of the sample)/A_540_ of the blank × 100 (2)

### 2.5. Anti-α-Glucosidase Activity Assay

Anti-α-glucosidase activity of WSCGDs was measured by the method of Doan et al. [41], with a slight modification in the buffer system (0.1 mM Tris-HCl buffer pH 7 was used to replace sodium phosphate buffer).

### 2.6. High Performance Liquid Chromatography Size Exclusion Chromatography (HPLC SEC) Analysis

To perform the HPLC SEC analysis, the sample was prepared at 1 mg/mL concentration in distilled water. A Hitachi Chromaster HPLC system (Hitachi, Tokyo, Japan) was assigned for the analysis using a KS-802 column and an UV-detector from Hitachi, Tokyo, Japan (the wavelength of 205 nm) under the following conditions: column temperature, 80 °C; flow rate, 0.6 mL/min; mobile phase, H_2_O. The standards were including *N*-acetyl-glucosamine, tri-*N*-acetyl chitotriose, hexa-*N*-acetyl chitohexaose, lysozyme (chicken egg white), and blue dextran (70 kDa).

### 2.7. Colloid Titration Analysis

An amount of WSC or WSCGD (20 mg) was disolved in 50 mL distilled water to make the diluted solution. The colloid titration analysis followed the method of Gullón et al. using potassium polyvinyl sulfate (PVSK, FUJIFILM Wako Pure Chemical Corporation, Osaka, Japan) and toluidine blue (Sigma-Aldrich, Taipei, Taiwan) [15].

### 2.8. Fourier Transform Infrared Spectroscopy (FTIR) Analysis

The FTIR analysis followed the method of Gullón et al. [15].

### 2.9. Proton Nuclear Magnetic Resonance (^1^H-NMR) Analysis

The sample was prepared in D_2_O at 5 mg/mL of concentration. The ^1^H-NMR analysis(Bruker Avance 600 MHz NMR spectrometer, Bruker, Billerica, Massachusetts, USA) was carried out at 600 MHz.

## 3. Results

### 3.1. Water-Soluble Chitosan–Glucose Derivatives’ (WSCGDs) Formation via Maillard Reaction

**Figure 1:**
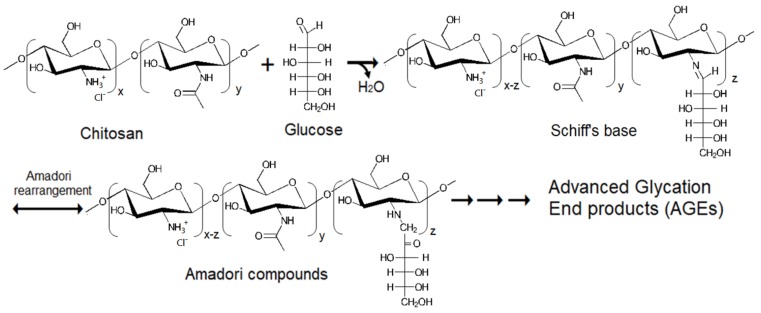
Maillard reaction of water-soluble chitosan (WSC) and glucose.

**Figure 2:**
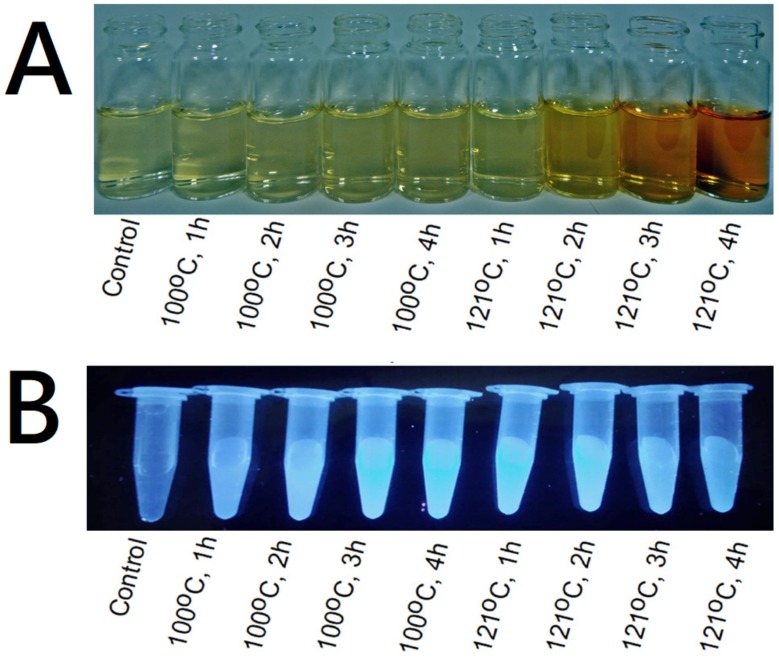
Photograph of WSC and WSCGDs under natural light (**A**) and ultraviolet light (**B**). All solutions had the same concentration of 1 mg/mL.

The Maillard reaction of chitosan and glucose was reported in other studies [15,16,17,18,19]. Consequently, the scheme of this reaction between WSC and glucose could be shown in Figure 1. The Maillard reaction can be easily observed by the absorbance at 294 nm (the formation of intermediates), and 420 nm (the formation of Melanoidins, final products of Maillard reaction) [15,16,17,18,19]. As shown in Table 1, absorbance at 294 nm of WSCGDs prepared at 100 °C was slightly increased after 2 h (0.078–0.109, respectively) compared to the control (0.037), whereas those from 121 °C expressed a strong increase from 0.215 (121 °C for 1 h) to 0.744 (121 °C for 4 h). This result indicated that heating temperature at 121 °C had a higher efficiency than 100 °C in the formation of intermediates. Additionally, absorbance at 420 nm of WSCGDs also resulted in a similar pattern. In detail, the highest increase in absorbance at 420 nm was observed at WSCGDs from heating temperature 121 °C (0.032–0.170, 1–4 h, respectively) in the comparison with those from 100 °C (0.015–0.025, 1–4 h, respectively) and the control (0.013). This result revealed that heating temperature at 121 °C also had higher efficiency than 100 °C in producing browning products. The development of Maillard reaction products were easily observed in Figure 2A, in which brown color clearly appeared in the case of 121 °C heating temperature. The ratio of A_294_/A_420_ expressed the stage of the reaction. The increase of A_294_/A_420_ indicated that the process was in the intermediate stage, whereas the decrease confirmed the final stage of the reaction [15]. In the case of 100 °C heating temperature, the A_294_/A_420_ ratio was still in an uptrend although there was no statistical difference between values from 1 h to 4 h. This result suggests that the main process of the Maillard reaction of WSC and glucose at 100 °C for 1–4 h was the formation of intermediates. Otherwise, the A_294_/A_420_ ratio of 121 °C heating temperature shows a clear decrease from 1 h to 4 h (6.737–4.379, respectively), indicating the final stage of the Maillard reaction. In short, the Maillard reaction of WSC and glucose could take place more effectively at 121 °C heating temperature. So far, the optimum heating temperature for the Maillard reaction of chitosan and glucose was observed at various points [15,19,23].

In the Maillard reaction, intermediates may express fluorescence ability [15]. As shown in Figure 2B, all the WSCGDs showed the fluorescence ability under UV light. This result confirmed that the Maillard reaction was being performed between water-soluble chitosan and glucose. A decrease in fluorescence ability of 121 °C at 3 h and 4 h could also be observed with the naked eye, indicating the final stage of the reaction, when the intermediates were transformed into final products.

### 3.2. Anti-Oxidant Activity

Free radicals cause some serious damage in cells as well as tissues. The injurious effect of free radicals can be prevented or reduced by anti-oxidants [41], which include chitosan (in low molecular weight) and its derivatives [5,14,17,19,20,22,23]. Table 2 shows the anti-oxidant activity of WSCDs using DPPH radical scavenging assay. All the WSCGDs exhibit higher anti-oxidant activity than native water-soluble chitosan (in maximum activity at 10 mg/mL of concentration and the IC_50_ value). The result indicates an increase in the anti-oxidant ability of WSCGDs with the increased heating time and reaction temperature. WSCGDs treated at 121 °C for 2, 3 and 4 h, which show the maximum anti-oxidant activity at 10 mg/mL (87.03–92.52%), also exhibited the highest ability in scavenging the DPPH radicals with the IC_50_ from 1.90–1.05 mg/mL. The native water-soluble chitosan showed low anti-oxidant activity when only 25.44% of activity was observed at 10 mg/mL; consequently, its IC_50_ could not be detected. This result was similar to other reports, which reveal that the low anti-oxidant activity of chitosan and glucose-conjugate was one of the potential ways to enhance the anti-oxidant activity of chitosan [17,19,20]. The mechanics of anti-oxidant activity of glucose-conjugated chitosan may relate to its hydrogen donating capacity [19].

### 3.3. Anti-α-Amylase Activity

Alpha-amylase catalyzes the hydrolysis reaction of dietary starch to release oligo- or di-saccharides, which are finally converted to glucose by the activity of α-glucosidase. Later on, free glucose is adsorbed by the gastrointestinal wall, resulting in an increased level of glucose in the blood (postprandial hyperglycemia) [43]. Delaying an increase in the postprandial glucose level in the blood can be done by blocking the action of α-amylase. In this study, anti-α-amylase activity of the WSC and WSCGDs were investigated in a range of concentration from 5 mg/mL to 20 mg/mL. All the WSC and WSCGDs exhibited a dose-dependence of anti-α-amylase activity (data not shown,). As shown in Table 3, the highest anti-α-amylase activity of the WSC and WSCGDs was found at 20 mg/mL of concentration with a range of activity 23.13%–56.56%. Only the WSCGDs from the 121 °C treatment for 3 h and 4 h expressed anti-α-amylase over 50%, whereas other treatments showed lower activity. Their IC50 value was also investigated, and found to generate values of 18.02 mg/mL (121 °C, 3 h) and 18.37 mg/mL (121 °C, 4 h). The anti-α-amylase activity of the WSC and its derivatives were rarely reported. Low molecular weight chitosans (1000 Da <, 1000–10.000 Da, and > 10.000 Da) were observed to exhibit low anti-α-amylase, with under 40% of maximum activity at 20 mg/mL of concentration, matching the result in this study [42]. Additionally, the results from this study promise potential improved anti-α-amylase activity of low molecular weight chitosan via conjugation reaction with reducing sugar as well as glucose.

### 3.4. Anti-α-Glucosidase Activity

Since anti-α-glucosidase activity is a strategic point in managing diabetes type II, the WSC and WSCGDs were also tested for this ability in a range 5–20 mg/mL of concentration. Similar to results of anti-α-amylase activity, all the WSC and WSCGDs also exhibited a dose-dependence of anti-α-glucosidase activity (data not shown). At 20 mg/mL of concentration, the anti-α-glucosidase activity of WSC and WSCGDs was observed at the value of 69.07–90.63%. WSCGDs from 121 °C for 3 h and 4 h showed the highest value of maximum activity (89.16% and 90.63%, respectively) in comparison with other treatments. As shown in Table 4, WSCGDs from the treatments at 121 °C can achieve a higher IC_50_ value of anti-α-glucosidase activity (7.09–5.72 mg/mL) than from the treatments at 100 °C and the control (10.17–8.71 mg/mL). The increase in anti-α-glucosidase activity of WSCGDs may relate to the present of Maillard reaction products. Hwang et al. reported that 2,4-bis (p-hydroxyphenyl)-2-butenal (a product from fructose–tyrosine Maillard reaction) expressed the best anti-α-glucosidase activity with the IC 50 value of 4.00 µg/mL [24]. Chitosan and its derivatives have demonstrated great ability in anti-diabetes; consequently, the research is still ongoing [46]. However, only several reports show the anti-α-glucosidase activity of chitosan [41,42,43]. As a result, this study may be a novel contribution to anti-α-glucosidase as well as anti-diabetes research on chitosan and chitosan derivatives.

By expressing the highest ability, WSCGDs from the 121 °C treatment for 3 h and 4 h may be considered as a strong candidate for anti-oxidant and anti-diabetes activities. Since there was no statistical difference between the activities (anti-oxidant, anti-α-amylase and anti-α-glucosidase) of 121 °C for 3 h and 4 h, WSCGD of 121 °C for 3 h was chosen for further investigation due to its shorter heating time.

### 3.5. HPLC SEC Analysis

**Figure 3:**
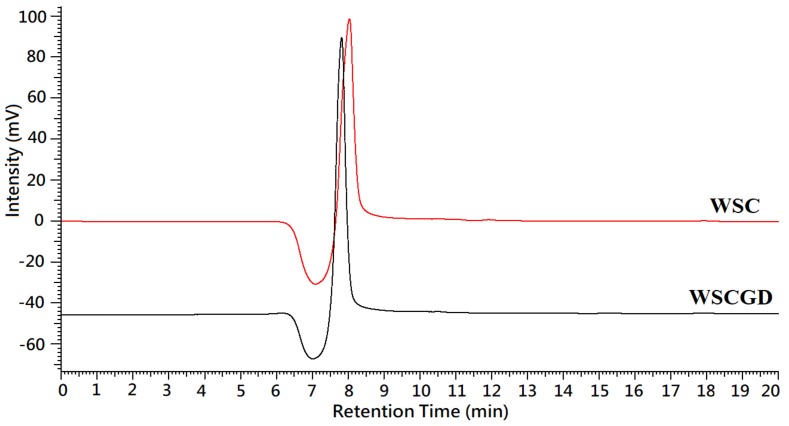
Typical high performance liquid chromatography size exclusion chromatography (HPLC SEC) profiles of WSC and WSCGD.

HPLC SEC analysis based on the KS-802 column was used to figure out the change in the molecular weight of WCS and WCSG. As shown in Figure 3, the peak of WCS was observed at 8.04 min, corresponding to a mass of 12.5 kDa, whereas the peak of WCSG was l located at 7.82 min (corresponding to a mass of 15.1 kDa). This result indicated that the conjugation between glucose and chitosan caused an increase in the molecular weight of chitosan. The molecular weight increase after performing the conjugations between glucose and chitosan in different molecular weights was also observed in several reports; for instance, an 8.3 kDa chitosan gave a rise to a glucose-conjugated chitosan with 12.4 kDa [15], or a 123 kDa chitosan to its derivative with 210 kDa molecular weight [23]. Additionally, there was dissimilarity in the width of the WSC and WSCG peaks; the WCSG peak revealed a narrower width than that of WSC. This result suggests there was reduced viscosity of WSCG in the comparison with WSC. The viscosity measurement also revealed that WSCGD showed a slight decrease in viscosity value in the comparison with WSC (2.010 ± 0.010 cp and 2.108 ± 0.008 cp, respectively). The reduction in viscosity of chitosan derivative was also observed by other reports [16].

### 3.6. Colloid Titration Analysis

As –NH_2_ groups of chitosan condense with carbonyl group of glucose in Maillard reaction, a decrease in degree of deacetylation (DD) of chitosan derivatives could be observed. Consequently, the decrease in DD of chitosan derivatives is also assigned to establish the degree of substitution (DS) [15,21]. In this study, colloid titration analysis based on PVSK was used to figure out the DD of native WSC and WSCGD. WSC was observed at 75.95 ± 2.72% of DD whereas that of WSCGD was significantly decreased to 41.32 ± 3.05%. Consequently, The degree of acetylation (DA) of WSC was estimated at 24.05% (approximately). This result suggests that the Maillard reaction of the WSC and glucose at 121 °C for 3 h possessed a DS value at 34.62 ± 2.78%. Several reports reveal that chitosan–glucose derivatives from chitosan with 90% of DD could possess a higher DS (over 50%) [15,21,47]. However, to our knowledge, DS of chitosan–glucose derivatives from chitosan with lower DD was rarely reported. In addition, molecular weight of chitosan and reaction conditions were also the key factors for synthesizing chitosan–glucose derivatives [15,48]. For this reason, the results of this study could be considered as a contribution to establishing chitosan–glucose derivatives as well as exploring their bio-activities.

### 3.7. FTIR Analysis

**Figure 4:**
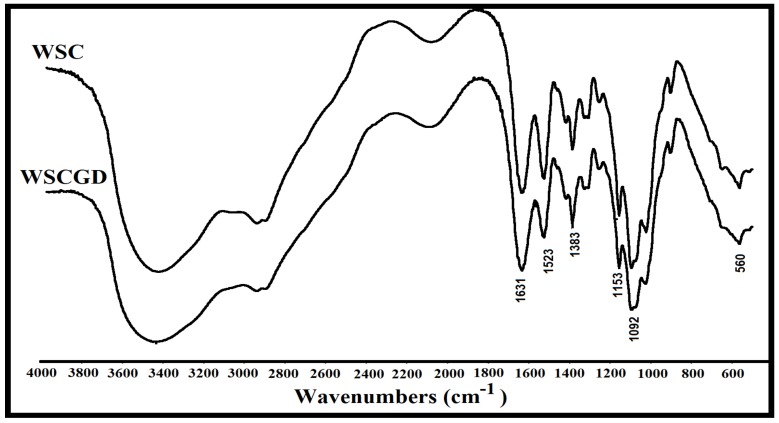
Typical FTIR profiles of WSC and WSCGD.

FTIR analysis was employed to figure out the chemical groups in WSC and WSCG. The peaks confirmed that the chitosan spectrum can be observed at 3440 cm^−1^ (–OH, –NH_2_), 2927 cm^−1^ (–CH), 1631 cm^−1^ (amide I), 1523 cm^−1^ (amide II), 1383 cm^−1^ (amide III) on both the WSC and WSCG patterns (Figure 4). The reduction of 1523 cm^−1^ band in the WSCGD spectrum, which was assigned to the amide group, suggested that a conjugation between the amine group of chitosan and carboxyl group of glucose was successfully performed [23]. The attachment of glucose to WSC also led to a decrease in the 1092 cm^−1^ band (assigned to C–O stretching vibration). Similar phenomena were also observed in other reports [15,16].

### 3.8. H-NMR Analysis

**Figure 5:**
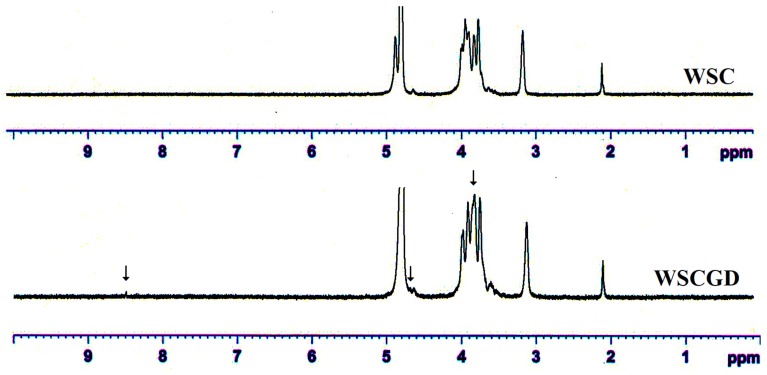
Typical ^1^H-NMR profiles of WSC and WSCGD. ↓ indicating the difference in ^1^H-NMR signal of WSCGD comparing to that of WSC.

^1^H-NMR analysis was used to investigate the structural changes on the WSC and WSCGD. As shown in Figure 5, the signals confirming the structure of chitosan in WSC and WSCGD spectra could be observed at δ 2.1 ppm (methyl proton on the acetyl group), δ 3.1 ppm (H–2 of GlcN ring), a multiplet of δ 3.7–4.0 ppm (chemical shifts of H–3, H–4, H–5, and H-6 in the glucose skeleton), and ppm δ 4.9 ppm (hydrogen on C–1) [15,18]. A strong signal at δ 4.8 ppm belonged to solvent (D_2_O). By comparing the ^1^H-NMR spectra, some alterations between WSC and WSCGD were found. A new signal at δ 8.5 ppm could be assigned for the –N=CH– group (Schiff’s base), an intermediate of the Maillard reaction. A new signal at δ 4.7 ppm may relate to the N-substitution of the NH_2_ groups [15]. The change of signals in a range of 3.8–4.0 ppm was also observed in the WSCGD spectra, indicating an alteration in the structure of chitosan.

## 4. Conclusions

In the present work, water-soluble chitosan–glucose derivatives (WSCGDs) were successfully produced via Maillard reaction under the heating temperatures 100 °C and 121 °C. WSCGDs possessed higher anti-oxidant and anti-diabetes (anti-α-amylase and anti-α-glucosidase) activities than native water-soluble chitosan. To our knowledge, this could be a novel report revealing the anti-α-amylase and anti-α-glucosidase abilities of glucose-conjugated chitosan derivatives. WSCGD from the treatment of 121 °C for 3 h was also investigated in regard to the structural characters by HPLC SEC, FTIR, colloid titration, ^1^H-NMR analyses to confirm the conjugation reaction. Showing stronger anti-oxidant and anti-diabetes activities, WSCGD might be an attractive candidate for future use in the medicine or health food fields.

## Figures and Tables

**Table 1:** Absorbance at 294 nm and 420 nm of water-soluble chitosan–glucose derivatives (WSCGDs) from different treatment times and temperatures *.

Treatment	A_294_	A_420_	A_294_/A_420_
Control	0.037 ± 0.001 ^g^	0.013 ± 0.001 ^g^	2.977 ± 0.363 ^e^
100 °C, 1 h	0.056 ± 0.059 ^fg^	0.015 ± 0.010 ^fg^	3.763 ± 0.415 ^d^
100 °C, 2 h	0.078 ± 0.003 ^f^	0.018 ± 0.001 ^f^	4.254 ± 0.083 ^d^
100 °C, 3 h	0.103 ± 0.002 ^e^	0.024 ± 0.000 ^e^	4.278 ± 0.087 ^cd^
100 °C, 4 h	0.109 ± 0.010 ^e^	0.025 ± 0.001 ^e^	4.420 ± 0.072 ^cd^
121 °C, 1 h	0.215 ± 0.055 ^d^	0.032 ± 0.001 ^d^	6.737 ± 0.372 ^a^
121 °C, 2 h	0.393 ± 0.089 ^c^	0.073 ± 0.015 ^c^	5.404 ± 0.089 ^b^
121 °C, 3 h	0.598 ± 0.083 ^b^	0.121 ± 0.003 ^b^	4.932 ± 0.057 ^bc^
121 °C, 4 h	0.744 ± 0.017 ^a^	0.170 ± 0.036 ^a^	4.379 ± 0.167 ^cd^

^*^ The measurement was carried out at 1 mg/mL of concentration. The letters a, b, c, d, e, f, and g in the same column represented for the statistical difference of means with *p* < 0.05 using Tukey-test. All data points in the table are mean and standard deviation.

**Table 2:** Anti-oxidant activity of WSC and WSCGDs.

Treatment	IC_50_ (mg/mL)	Maximum Activity * (%)
Control	ND	25.44 ± 1.39 ^e^
100 °C, 1 h	13.94 ± 0.75 ^a^	45.14 ± 3.12 ^d^
100 °C, 2 h	9.69 ± 0.56 ^b^	50.71 ± 1.25 ^cd^
100 °C, 3 h	8.75 ± 0.75 ^b^	52.37 ± 1.39 ^c^
100 °C, 4 h	8.38 ± 0.72 ^b^	55.11 ± 1.63 ^c^
121 °C, 1 h	4.99 ± 0.33 ^c^	68.33 ± 2.38 ^b^
121 °C, 2 h	1.90 ± 0.21 ^d^	87.03 ± 1.94 ^a^
121 °C, 3 h	1.28 ± 0.16 ^d^	92.52 ± 4.11 ^a^
121 °C, 4 h	1.05 ± 0.19 ^d^	92.69 ± 1.23 ^a^

^*^ Maximum activity of all WSCGDs was determined at the same concentration of 10 mg/mL. ND: not detected. The letters a, b, c, d, e in the same column represent the statistical difference of means with *p* < 0.05 using Tukey-test. All data points in the table are mean and standard deviation. The anti-oxidant activity assay was conducted at 20 °C for 20 min.

**Table 3:** Anti-α-amylase activity of the WSC and WSCGDs.

Treatment	IC_50_ (mg/mL)	Maximum Activity * (%)
Control	ND	23.13 ± 3.64 ^c^
100 °C, 1 h	ND	25.71 ± 4.06 ^bc^
100 °C, 2 h	ND	25.62 ± 4.78 ^bc^
100 °C, 3 h	ND	38.25 ± 4.39 ^bc^
100 °C, 4 h	ND	27.13 ± 6.79 ^bc^
121 °C, 1 h	ND	31.43 ± 6.23 ^bc^
121 °C, 2 h	ND	36.79 ± 4.67 ^b^
121 °C, 3 h	18.02 ± 0.88	56.56 ± 4.51 ^a^
121 °C, 4 h	18.37 ± 1.33	56.07 ± 5.67 ^a^

^*^ Maximum activity of all WSCGDs was determined at the same concentration of 20 mg/mL. ND: not detected. The letters a, b, c, d, e in the same column represent the statistical difference of means with *p* < 0.05 using the Tukey-test. All data points in the table are mean and standard deviation. The anti-α-amylase activity of the WSC and WSCGDs were conducted at 20 °C for 20 min.

**Table 4:** Anti-α-glucosidase activity of WSCGs.

Treatment	IC_50_ (mg/mL)	Maximum Activity * (%)
Control	10.04 ± 0.45 ^a^	69.07 ± 2.04 ^d^
100 °C, 1 h	10.17 ± 0.64 ^a^	70.73 ± 3.45 ^d^
100 °C, 2 h	9.33 ± 0.07 ^ab^	72.02 ± 2.56 ^cd^
100 °C, 3 h	9.13 ± 0.39 ^ab^	74.28 ± 2.64 ^cd^
100 °C, 4 h	8.71 ± 0.39 ^b^	72.92 ± 0.87 ^bcd^
121 °C, 1 h	7.09 ± 0.14 ^c^	77.42 ± 1.87 ^bc^
121 °C, 2 h	6.15 ± 0.33 ^cd^	80.65 ± 2.17 ^b^
121 °C, 3 h	5.72 ± 0.36 ^d^	89.16 ± 2.52 ^a^
121 °C, 4 h	5.85 ± 0.33 ^d^	90.63 ± 0.56 ^a^

^*^ Maximum activity of all WSCGDs was determined at the same concentration of 20 mg/mL. ND: not detected. The letters a, b, c, d, e in the same column represent the statistical difference of means with *p* < 0.05 using the Tukey-test. All data points in the table are mean and standard deviation. The anti-α-glucosidase activity of the WSC and WSCGDs were conducted at 20 °C for 20 min.

## Supplementary Materials

The following are available online at https://www.mdpi.com/2073-4360/11/10/1714/s1, Figure S1: HPLC profile of chitosan hydrolysate. The hydrolysis reaction was performed to the following conditions: 0.1 mg/mL of WSC, 40 U of Streptomyces speibonae TKU048 chitinase, pH 7 (using 20 mM Tris HCl buffer), 50oC of temperature, and 7 days of incubation time. The condition for HPLC analysis was including NH2-50 4E column, 70/30 (CH3CN/potassium phosphate buffer pH 7.5) of solvent, 0.9 mL/min of flow rate, 40oC of column temperature, 20 μL of sample volume, UV detector 190 nm. The result confirms that the main monomer components of the WSC was N-acetyl-D-glucosamine (peak 1) and glucosamine (peak 2), Figure S2: Solubility profile of WSC under different pH points.
