# Supplementary material for: Anti-Oxidant and Anti-Diabetes Potential of Water-Soluble Chitosan–Glucose Derivatives Produced by Maillard Reaction
Source: Polymers (Basel). 2019 Oct 18;11(10):1714. doi: 10.3390/polym11101714 (PMC6836137; doi:10.3390/polym11101714)
Supplement: Supplementary file 1 [file polymers-11-01714-s001.pdf]

Supplementary Materials

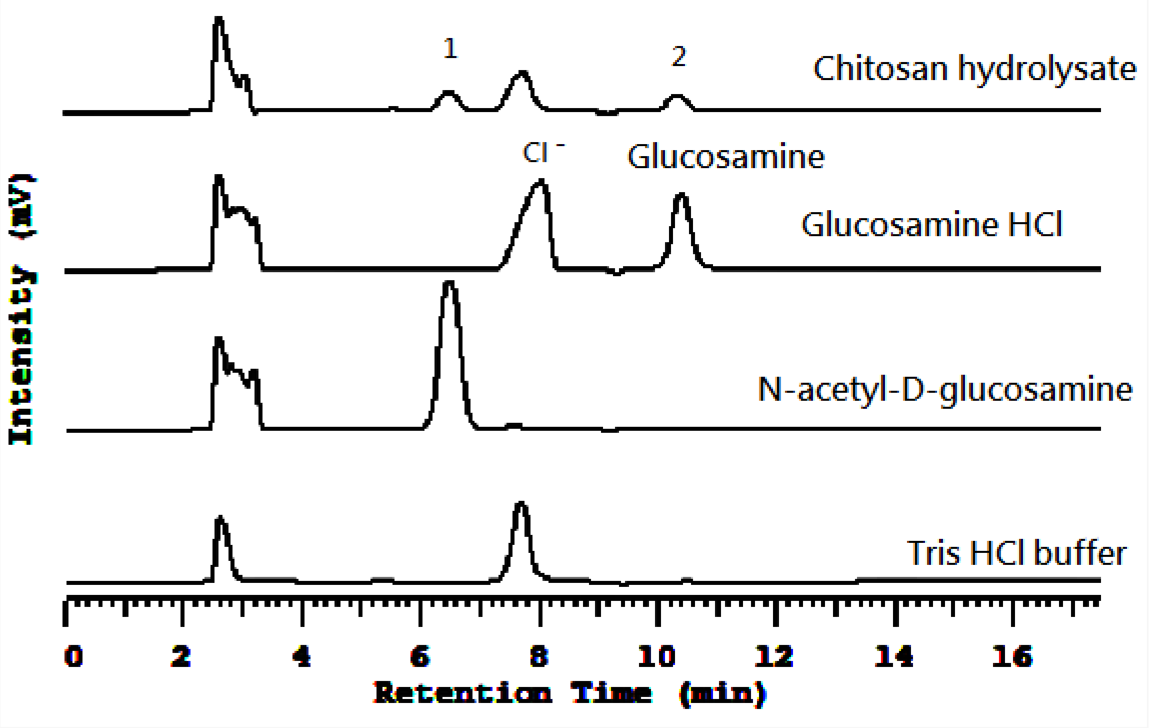

Figure S1. HPLC profile of chitosan hydrolysate. The hydrolysis reaction was performed to the following conditions: 0.1 mg/mL of WSC, 40 U of *Streptomyces speibonae* TKU048 chitinase, pH 7 (using 20 mM Tris HCl buffer), 50°C of temperature, and 7 days of incubation time. The condition for HPLC analysis was including NH_2-50 4E column, 70/30 (CH_3CN/potassium phosphate buffer pH 7.5) of solvent, 0.9 mL/min of flow rate, 40°C of column temperature, 20 μ L of sample volume, UV detector 190 nm. The result confirms that the main monomer components of the WSC was N-acetyl-D-glucosamine (peak 1) and glucosamine (peak 2).

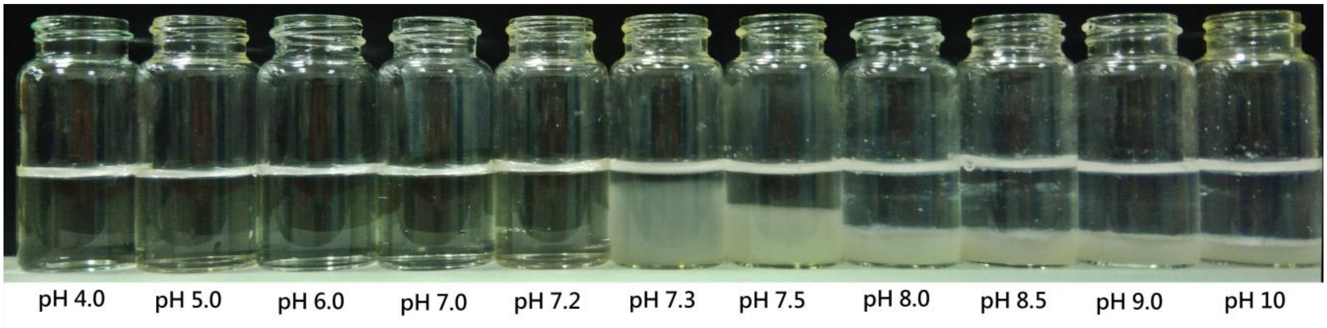

Figure S2. Solubility profile of WSC under different pH points.
